# What is associated with painful polyneuropathy? A cross-sectional analysis of symptoms and signs in patients with painful and painless polyneuropathy

**DOI:** 10.1097/j.pain.0000000000003310

**Published:** 2024-07-03

**Authors:** Janne Gierthmühlen, Nadine Attal, Georgios Baskozos, Kristine Bennedsgaard, David L. Bennett, Didier Bouhassira, Geert Crombez, Nanna B. Finnerup, Yelena Granovsky, Troels Staehelin Jensen, Jishi John, Lieven Nils Kennes, Helen Laycock, Mathilde M.V. Pascal, Andrew S.C. Rice, Leah Shafran-Topaz, Andreas C. Themistocleous, David Yarnitsky, Ralf Baron

**Affiliations:** a Interdisciplinary Pain Unit, Department of Anesthesiology and Surgical Intensive Care Medicine, University Hospital of Schleswig-Holstein, Campus Kiel, Germany; b Division of Neurological Pain Research and Therapy, Department of Neurology, University Hospital of Schleswig-Holstein, Campus Kiel, Germany; c Inserm U987, APHP, CHU Ambroise Pare, UVSQ, Paris-Saclay University, Boulogne-Billancourt, France; d The Nuffield Department of Clinical Neuroscience, University of Oxford, Oxford, United Kingdom; e Department of Clinical Medicine, Danish Pain Research Center, Aarhus University, Denmark; f Department of Experimental Clinical and Health Psychology, Ghent University, Ghent, Belgium; g Department of Neurology, Aarhus University Hospital, Aarhus, Denmark; h Department of Neurology, Rambam Health Care Campus, Haifa, Israel; i Faculty of Medicine, Technion, Haifa, Israel; j Department of Economics and Business Administration, University of Applied Sciences Stralsund, Stralsund, Germany; k Pain Research, Department of Surgery and Cancer, Faculty of Medicine, Imperial College, London, United Kingdom

**Keywords:** Painful neuropathy, Painless neuropathy, Neuropathic pain, Machine learning, Pain prediction, PROMs, QST

## Abstract

It is still unclear how and why some patients develop painful and others painless polyneuropathy. The aim of this study was to identify multiple factors associated with painful polyneuropathies (NeuP). A total of 1181 patients of the multicenter DOLORISK database with painful (probable or definite NeuP) or painless (unlikely NeuP) probable or confirmed neuropathy were investigated clinically, with questionnaires and quantitative sensory testing. Multivariate logistic regression including all variables (demographics, medical history, psychological symptoms, personality items, pain-related worrying, life-style factors, as well as results from clinical examination and quantitative sensory testing) and machine learning was used for the identification of predictors and final risk prediction of painful neuropathy. Multivariate logistic regression demonstrated that severity and idiopathic etiology of neuropathy, presence of chronic pain in family, Patient-Reported Outcomes Measurement Information System Fatigue and Depression T-Score, as well as Pain Catastrophizing Scale total score are the most important features associated with the presence of pain in neuropathy. Machine learning (random forest) identified the same variables. Multivariate logistic regression archived an accuracy above 78%, random forest of 76%; thus, almost 4 out of 5 subjects can be classified correctly. This multicenter analysis shows that pain-related worrying, emotional well-being, and clinical phenotype are factors associated with painful (vs painless) neuropathy. Results may help in the future to identify patients at risk of developing painful neuropathy and identify consequences of pain in longitudinal studies.

## Abstract 2

The study makes a first step to predict the probability of painful neuropathy. Knowing individual risks might help to prevent the onset of pain.

## 1. Introduction

Prevalence of chronic pain with neuropathic characteristics ranges between 7% and 10% in the general population, with polyneuropathy being a common cause.^68^ Over 100 different causes of polyneuropathy have been identified, including diabetic neuropathies (estimated prevalence of 11%-30%,^1,4,18^) and multiple nondiabetic conditions (ie, alcoholic, associated with endocrine or immune diseases, hereditary, drug or toxin-associated, malignant etc.^31,45,58^). Painful neuropathies can be grouped into symmetrical polyneuropathies, ie, diseases affecting many nerves simultaneously, typically in a length-related glove-and-stocking distribution, asymmetrical neuropathies with mono- or multiplex distribution, or processes affecting plexuses or single nerves.^58^

It is still unclear why some patients develop painful and others painless polyneuropathies. Because there is no definitive single or overall composite factor that explains individual pain vulnerability in polyneuropathy, surrogate markers potentially associated with mechanisms for pain can be used. One approach is to assess the individual functioning of the somatosensory nervous system (loss of function/gain of function of nociceptive and non-nociceptive sensory modalities) using quantitative sensory testing (QST). The German Research Network on Neuropathic Pain has developed a standardized QST battery that investigates different afferent nerve fiber functions and their central pathways^53^ to address the hypothesis that different clinical signs reflect different underlying mechanisms.^5^ Hierarchical cluster analysis has shown that these mechanism-related sensory profiles can be classified into 3 groups of neuropathic pain profiles: (1) sensory loss, (2) thermal hyperalgesia, and (3) mechanical hyperalgesia.^6^

However, signs and symptoms of polyneuropathy are not restricted to sensory dysfunction but may also include motor and autonomic disturbances.^12^ Although some characteristic symptoms may be indicative of certain etiologies of polyneuropathy especially genetic causes, the clinical phenotype usually varies between patients even with the same etiology.^41,64^ There is likely a bidirectional relationship between the initiation and maintenance of pain and factors such as genetic variants,^56,57,78^ sociodemographic factors,^8,19^ physical activity,^21,50^ multiple psychosocial,^37,66^ and lifestyle factors,^3,59^ including sleep problems, life satisfaction, and adverse childhood events, as well as pain-related worrying^65^ and emotional functioning (anxiety or depression^39,65^).

The study aimed to identify the various factors that differentiated patients with painful neuropathy from those with painless neuropathy. Therefore, data from self-reported measures, clinical findings, and QST from the Dolorisk database^46^ were analyzed with multiple regression analyses and machine learning (ML) models.

## 2. Methods

### 2.1. Standard protocol approvals, registrations, and patient consents

The study was approved by the local ethics committees at each participating center. Informed consent was obtained from all participants according to the Declaration of the World Medical Association. The study was registered at clinicaltrials.gov (NCT04888455).

### 2.2. Study set-up

The DOLORISK consortium recruited patients with neuropathy >18 years of age from tertiary centers between 2016 and 2019. The DOLORISK study protocol has been described by Pascal et al.^46^ In short, patients attending tertiary centers for treatment or further evaluation of symptoms of neuropathy underwent phenotyping by different questionnaires and clinical neurological examination including QST according to the German Research Network on Neuropathic Pain,^53^ using a shortened protocol (see below). Questionnaires assessed demographic data, pain characteristics, health status, emotional well-being, personality, and lifestyle. Only data of the cross-sectional measures were used for this analysis.

### 2.3. Patients sample

Data of 1550 patients of the DOLORISK database were screened, and all records extracted that fulfilled the criterion of presence of probable (presence of a combination of symptoms and signs of neuropathy include any 2 or more of the following: neuropathic symptoms, decreased distal sensation, or unequivocally decreased or absent ankle reflexes) or confirmed (presence of an abnormality of nerve conduction or validated measure of small fiber neuropathy with class 1 evidence with corresponding symptoms) neuropathy according to the Toronto Consensus Panel on Diabetic Neuropathy.^20,63^ Patients were then further divided into those with painful and painless neuropathy according to the NeuPSIG algorithm^23^: Patients with probable or definite neuropathic pain were classified as painful neuropathy, and those with unlikely neuropathic pain and without neuropathic pain classified as painless neuropathy. Note that in those patients with painless neuropathy, patients may still experience pain at other locations (ie, headache or musculoskeletal joint pain). Patients with possible neuropathic pain were excluded from analysis because of lack of certainty for neuropathic pain. Also, patients with skin lesions or dermatological disorders in the areas to be tested upon QST, with any painful or neurological comorbidity that could otherwise influence testing results such as vascular disease, radiculopathy, spinal canal stenosis etc. Inclusion was restricted to patients with distal symmetrical polyneuropathy to make the investigated patient sample as homogenous as possible.

### 2.4. Questionnaires

#### 2.4.1. Demographic data and lifestyle

Age, sex, body mass index (BMI), ethnicity, years in education, family history of chronic pain (whether a first-degree relative report suffering from pain for more than 3 months), etiology of neuropathy, presence of adverse childhood experiences and hospital admissions, smoking, and alcohol habits were assessed. Harmful alcohol consumption was defined as more than 7 drinks per week of 0.5 to 0.6L beer or 0.25 to 0.3L wine or 25 mL spirits for males and more than 3.5 drinks/week of 0.25 to 0.3 L beer or 0.125 to 0.15L wine or 12.5 mL spirits for females according to >140 g (males) resp. >70 g (females) alcohol per week as limit recommended by the German Society for Nutrition.

#### 2.4.2. Pain characteristics

In the group of patients with pain, pain duration, pain course, and chronic pain grade (CPG) according to Von Korff^71^ were assessed. Pain severity was assessed by calculating the average of the 4 pain items from Brief Pain Inventory (BPI^14^): “Pain at its worst in the last 24 hours,” “Pain at its least in the last 24 hours,” “BPI Pain on the average in the last 24 hours,” “Pain right now” upon the BPI.

Severity of neuropathic pain was assessed using the Neuropathic Pain Symptom Inventory.^9^

#### 2.4.3. Emotional well-being

The Patient-Reported Outcomes Measurement Information System (PROMIS^13^) questionnaires were used to assess anxiety (PROMIS Short Form v1.0—Anxiety 4a or 6a^48^), depression (PROMIS Short Form v1.0—Depression 4a or 6a^48^), sleep disturbances (PROMIS Short Form v1.0 Sleep Disturbance 4a or 6a^11^), and fatigue (PROMIS Short Form v1.0—Fatigue 4a, 6a, or 8a^36^). Items of these self-report questionnaires have 5 response options ranging from 1 to 5 (1 = not at all, 5 = very much). Sum scores of each scale are converted into a T-Score, resulting in a standardized score with a mean of 50 and a SD of 10 in a reference population. Values > 60 are considered above average. Higher scores reflect problem severity. For example, a T-score of 60 on the depression scale is indicative of a moderate depressed mood.

#### 2.4.4. Personality

The Ten-Item Personality Inventory (TIPI) evaluates 5 personality dimensions (extraversion, agreeableness, conscientiousness, emotional stability, and openness to experience) with 2 items each. The TIPI norms are based on data collected at https://gosling.psy.utexas.edu/scales-weve-developed/ten-item-personality-measure-tipi/.^29^ Higher values are associated with more extraversion, agreeableness, conscientiousness, emotional stability, and openness to experience, respectively.

Of these personality dimension, we aimed to explore the role of emotional stability in more depth, because this personality dimension has been found to be related to the reporting of health complaints and pain.^15,74^ For that reason, we also used the 10 item Emotional Stability Scale from the International Personality Item Pool (IPIP^28^). Patients' answer to what extent each item describes themselves using a 5 point scale (1 = “very inaccurate” and 5 = “very accurate”). A sum score is calculated. For the interpretation of individual scores, the mean and SD for a sample of persons (same sex and particular age range) is calculated. Scores within one-half SD of the mean can be interpreted as “average,” outside that range as “low” or “high.”^33^

Pain-related worrying was recorded through the Pain Catastrophizing Scale (PCS^16,62,75^). It consists of 13 statements describing different thoughts and feelings that may be associated with pain. These statements are to be rated on a 5-point Likert scale (ranging from 0 “not at all” to 4 “all the time”), a total score is obtained by scoring all the items. Higher scores indicate greater pain-related worrying. A total score of 30 is considered a relevant level of pain-related worrying, representing the 75th percentile of PCS scores in clinic samples of patients with chronic pain.

### 2.5. Clinical examination—severity of neuropathy

During clinical examination, nerve conduction tests and/or skin biopsy were performed to confirm the presence of a length dependent neuropathy using the algorithm of Tesfaye et al.^63^ as mentioned above. Upon clinical examination, severity of neuropathy was quantified with the Toronto Clinical Scoring System (TCSS^10^). The TCSS consists of 6 questions for presence of typical symptoms of polyneuropathy and an investigational part that examines muscle tendon reflexes of the lower extremities as well as reaction to different sensory stimuli (pinprick, temperature, vibration, light touch, proprioception) separate on both lower extremities. Two points are awarded for a missing reflex, one point for a weakened reflex, and 0 points for a normal reflex. Abnormal sensory examinations are awarded 1 point, normal ones 0 points. The maximum score is 1 for a simultaneous abnormal sensory sensation on the right and left foot.

Score may range from 0 to 19 (the higher the score, the more symptoms and abnormal findings upon examination). Because the TCSS includes a question for presence of pain that separated the 2 study groups anyway, we excluded this question from the total TCSS score resulting in a possible total score of 18. The TCSS total score was not calculated when one or more items were missing.

### 2.6. Quantitative sensory testing

All sites underwent strict quality control^69^ and an analysis of heterogeneity showed that the database could be analyzed as a homogenous dataset.^70^ Quantitative sensory testing was performed unilaterally in the most affected area, and the procedure described by Rolke et al.^53^ was followed with some exceptions because of time reasons: First, in case of proven pathological small fiber function, ie, pathological sensory loss detected by warm detection threshold and/or cold detection threshold thermal sensory limen was not be performed additionally. Second, only 2 repetitions for each stimulus were performed upon stimulus-response function for the assessment of mechanical pain sensitivity and dynamic mechanical allodynia. Before the start of the study, each center was trained for use of the protocol.

For evaluation of individual QST measurements, data were compared to a reference database of healthy controls^40,47,53^ and *z*-scores calculated. *Z*-Score (mean = 0, SD = 1) values indicate hypo- or hyperfunction of the subject's sensitivity for each parameter as compared with the mean of age- and sex-matched controls. The 95% confidence interval of controls is between −1.96 and +1.96. *Z*-values above “0” are indicative of hyperfunction, that is, patients are more sensitive to the tested parameter compared to controls (lower thresholds). *Z*-scores below “0” are indicative of hypofunction and, therefore, a loss of or lower sensitivity of the patient compared to controls (higher thresholds). To determine the sensory phenotype (sensory loss, thermal hyperalgesia, mechanical hyperalgesia) of each patient, we used the algorithm proposed by Baron et al.^6^

### 2.7. Statistical analysis

#### 2.7.1. Descriptive statistics

The distribution of variables was investigated separately for the 2 subgroups painful/painless neuropathy. Continuous variables are expressed as mean values ± SD. Categorical data are presented by absolute frequencies and/or percentages.

#### 2.7.2. Inferential statistics and predictive modelling

To identify the most powerful variables associated with painful polyneuropathy and to classify patients according to these variables, multivariate logistic regression (MLR) as well as random forest were conducted—for further details, see below.

#### 2.7.3. Multivariate logistic regression

A MLR including all variables (age, sex, BMI, family history of chronic pain, PROMIS anxiety, depression, fatigue and sleep T-Scores, previous traumatic events and hospital stays, TIPI subscores for extraversion, agreeableness, conscientiousness, emotional stability, openness, emotional stability score, PCS total score, TCSS total score, etiology of neuropathy, years of education, QST Cluster, as well as smoking and alcohol habits) was performed to investigate their influence on the pain status (painful or painless neuropathy). Because of missing values, missing imputation was performed by multivariate imputation by chained equations (MICE). Multiple (m = 25) complete data sets were derived by chained equations to account for uncertainty because of the missing information. Multivariate logistic regression models were applied on all complete data sets to determine final results by aggregating individual results according to Rubin rule.^55,67^

To estimate performance of prediction for unseen data, the “Leave-one-out”-method was used. For each participant, the logistic regression model was derived based on the remaining 1180 subjects and the resulting model used to predict the probability of painful neuropathy for the subject, who was left out during the training. If this probability was above 50%, the participant was classified as “painful neuropathy,” otherwise as “painless neuropathy.” This procedure was repeated for all 1181 subjects. The relative frequency of correct classifications is referred to as the accuracy. To make results comparable to the ML procedure random forest, this and other standard ML-performance metrics (accuracy, balanced accuracy, F1, and Kappa) were derived. To correct for imbalanced data (painful neuropathy: 843, painless neuropathy: 338), additionally to accuracy, the balanced accuracy and F1 statistics were derived. The balanced accuracy is the mean of the 2 relative frequencies of positive and negative cases identified correctly. The F1 statistics, commonly used in ML in case of imbalanced data, is defined as:

F1=2×(recall×precision)/(recall+precision)

Additionally, the Kappa-coefficient was determined to assess the level of agreement between predictions and true neuropathy status (painful/painless), correcting for random agreement.

#### 2.7.4. Machine learning

As a second method, ML, more specifically random forest, was used for the identification of predictor variables and risk estimation of painful neuropathy. The advantage of random forest compared to MLR with MICE is that random forest also recognizes nonlinear relationships.

A random forest with 500 trees was applied on the data. The 500 random samples of the usual 63.2% of observations were used to fit 500 decision trees. The remaining observations form the out-of-bag set and enable assessing performance on unseen data similar to the leave-one-out technique described above. Additionally, variable importance was assessed to determine how important different variables are for classifying the neuropathy status correctly. As the regular classification and regression trees are biased toward continuous variables and variables with many categories, unbiased conditional inference trees were used to account for the situation of different variable types.^32^ The same 4 performance metrics as for logistic regression were assessed for random Forest (ie, accuracy, balanced accuracy, F1, and Kappa).

#### 2.7.5. General

*P* < 0.05 was considered statistically significant; thus, the significance level was not adjusted for multiplicity. The statistical analyses were conducted using the statistical software R.

## 3. Results

**Figure 1.**
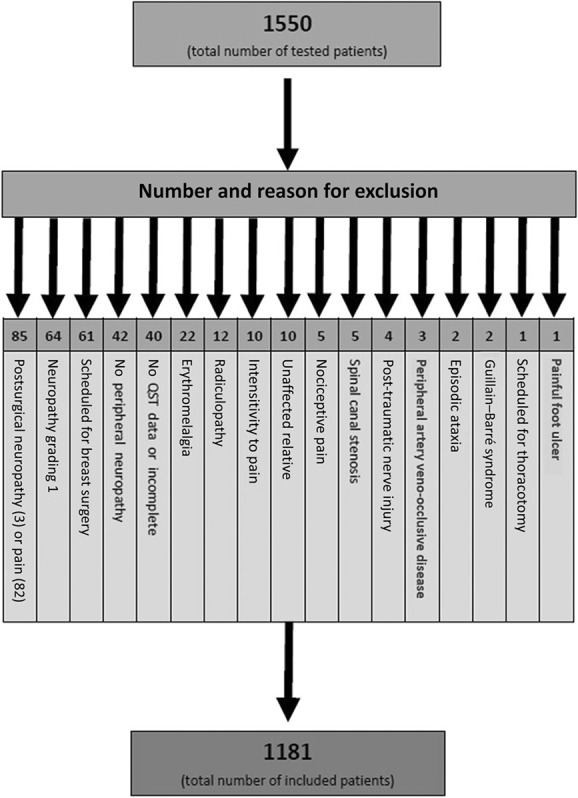
STROBE flowchart of included patients from the DOLORISK database. GBS, Guillan–Barré syndrome; PAVK, Peripheral artery disease; STROBE, Strengthening the Reporting of Observational studies in Epidemiology.

Data from 1181 participants were included in the analysis (Fig. 1). Approximately 2 thirds of patients were males. A total of 1061 (89.8%) had a confirmed neuropathy, 120 (10.2%) a probable neuropathy according to Tesfaye et al.^63^ A total of 843 (71.4%) of patients had a painful neuropathy (see also Tables 1–3), and 338 (28.6%) patients had a painless neuropathy (Tables 2 and 3).

**Table 1:** Pain characteristics of patients with painful neuropathy (n = 843).

BPI Pain Severity (mean ± SD [range]), n = 685	4.1 ± 2.4 (0-10)
NPSI total score (mean ± SD [range]), n = 762	29.6 ± 20.9 (0-95)
Pain course (n = 769), n (%)	
Persistent pain with slight fluctuations	198 (23.5%)
Persistent pain with pain attacks	316 (37.4%)
Pain attacks without pain between them	255 (30.2%)
Duration of pain (n = 792), n (%)	
Less than 1 month	9 (1.1%)
1-3 months	8 (0.9%)
3-12 months	64 (7.6%)
1-5 years	371 (44.0%)
More than 5 years	340 (40.3%)
Chronic pain grade (n = 509), n (%)	
Low disability—low intensity	220 (26.1%)
Low disability—high intensity	158 (18.7%)
High disability—moderately limiting	58 (6.9%)
High disability—severely limiting	73 (8.7%)

Note that both, BPI and NPSI, ask for a rating of pain within the last 24 hours; thus, it is possible to have a sum score of “0” (in case of presence of only intermittent pain that has not been present within the last 24 hours).

BPI, Brief Pain Inventory; NPSI, Neuropathic Pain Symptom Inventory.

**Table 2:** Clinical and demographic characteristics of patients with painful and painless neuropathy.

	All (n = 1181)	Painful neuropathy (n = 843)	Painless neuropathy (n = 338)	*P*
Age (y; mean ± SD [range])	65.8 ± 12.0 (19-92)	64.9 ± 12.4 (19-92)	68.1 ± 10.6 (19-87)	**<0.0001**
Sex, n (%)				
Males	776 (65.7%)	558 (66.2%)	218 (64.5%)	0.63
Females	405 (34.3%)	285 (33.8%)	120 (35.5%)	
BMI (mean ± SD [range])	29.1 ± 5.8 (14.9-67.9)	29.3 ± 5.9 (14.9-67.9)	28.6 ± 5.6 (18.8-58.1)	**0.049**
Ethnicity, n (%)	1001 (84.8%)	697 (82.7%)	304 (89.9%)	0.26
Asian	55 (4.7%)	41 (4.9%)	14 (4.1%)	
Black	33 (2.8%)	27 (3.2%)	6 (1.8%)	
White	992 (76.4%)	620 (73.6%)	282 (83.4%)	
Other	11 (0.9)	9 (1.1%)	2 (0.6%)	
Etiology of polyneuropathy, n (%)	1170 (99.1%)	839 (99.5%)	331 (97.9%)	**<0.0001**
Diabetic	872 (73.8%)	585 (69.4%)	287 (84.9%)	
Chemotherapy induced	55 (4.7%)	33 (3.9%)	22 (6.5%)	
Idiopathic	196 (16.6%)	179 (21.2%)	17 (5.0%)	
Other*	47 (4.0%)	42 (5.0%)	5 (1.5%)	
Toronto Total Score, n (%)† Mean ± SD (range)	1175 (99.5%) 9.36 ± 4.22 (0-18)	839 (99.5%) 9.89 ± 4.19 (0-18)	336 (99.4%) 8.03 ± 3.82 (1-18)	**<0.001**
QST cluster, n (%)	1173 (99.3%)	840 (99.6%)	333 (98.5%)	0.98
Mechanical hyperalgesia	303 (25.7%)	218 (25.9%)	85 (25.1%)	
Sensory loss	651 (55.1%)	466 (55.3%)	185 (54.7%)	
Thermal hyperalgesia	219 (18.5%)	156 (18.5%)	63 (18.6%)	

* Etiologies of other neuropathies include alcoholic neuropathy, Vit. B12-deficiency, hereditary neuropathy.

† For the Toronto Total Score, the question for presence of pain was excluded from the total score resulting in a maximum score of 18 instead of 19.^43^

QST, quantitative sensory testing.

*P* (significance) refers to painful vs painless neuropathy.

**Table 3:** Questionnaire results of patients.

	All (n = 1181)	Painful neuropathy (n = 843)	Painless neuropathy (n = 338)	*P*
Years in education, n (%) Mean ± SD (range)	723 (61.2%) 13.4 ± 4.7 (3-74)	522 (61.9%) 13.3 ± 5.0 (3-74)	201 (59.5%) 13.8 ± 3.7 (3-28)	0.14
Current or previous alcohol misuse, n (%)*	758 (64.2%)	550 (65.2%)	208 (61.1%)	**0.0022**
No	663 (56.1%)	494 (58.6%)	169 (50%)	
Yes	95 (8.0%)	56 (6.6%)	39 (11.5%)	
Smoking yes/no, n (%)	756 (64.1%)	550 (65.2%)	206 (76.9%)	0.164
Packyears; (mean ± SD [range])	11.2 ± 24.8 (0-247.5)	12.2 ± 26.4 (0-247.5)	8.5 ± 19.9 (0-171.5)	**0.0395**
Family history of chronic pain, n (%)	1069 (90.5%)	748 (88.7%)	321 (95%)	**<0.001**
No	819 (76.6%)	531 (71.0%)	288 (89.7%)	
Yes	250 (23.4%)	217 (29.0%)	33 (10.3%)	
Previous traumatic events, n (%)	739 (62.6%)	534 (63.3%)	205 (60.7%)	0.36
None	514 (69.6%)	363 (68.0%)	151 (73.7%)	
Yes, 1	137 (18.5%)	105 (19.6%)	32 (15.6%)	
Yes, 2	40 (5.4%)	32 (6.0%)	8 (3.9%)	
Yes, more than 2	48 (6.5%)	34 (6.4%)	14 (6.8%)	
Long hospital periods before the age of 18, n (%)	738 (62.5%)	532 (63.1%)	206 (61%)	0.48
No	636 (86.2%)	455 (85.5%)	181 (87.9%)	
Yes	102 (13.8%)	77 (14.5%)	25 (12.1%)	
PROMIS depression, n (%)	77 (48.9%)	408 (48.4%)	169 (50%)	
T-score (mean ± SD [range])	50.0 ± 9.7 (38.2-81.3)	52.0 ± 9.7 (38.2-81.3)	45.3 ± 7.9 (38.2-68.7)	**<0.001**
Abnormal result, n, (%)	89 (15.4%)	82 (20.1%)	7 (4.1%)	**<0.001**
PROMIS anxiety, n (%)	520 (44%)	357 (42.3%)	163 (48.2%)	
T-score (mean ± SD [range])	49.4 ± 10.6 (37.1-83.1)	51.1 ± 11.0 (37.1-83.1)	45.6 ± 8.6 (37.1-70.8)	**<0.001**
Abnormal result, n (%)	79 (15.2%)	71 (19.9%)	8 (4.9%)	**<0.001**
PROMIS sleep, n (%)	571 (48.3%)	404 (47.9%)	167 (49.4%)	
T-score (mean ± SD, [range])	50.9 ± 9.7 (28.9-76.5)	52.3 ± 9.7 (28.9-76.5)	47.4 ± 8.6 (28,9-76.5)	**<0.001**
Abnormal result, n (%)	91 (15.9%)	79 (19.6%)	12 (7.2%)	**<0.001**
PROMIS fatigue, n (%)	439 (37.2%)	342 (40.6%)	97 (28.7%)	
T-score (mean ± SD, [range])	55.81 ± 10.9 (40.7-78.3)	57.8 ± 10.4 (40.7-78.3)	49.0 ± 9.6 (40.7-77.0)	**<0.001**
Abnormal result, n (%)	174 (39.6%)	159 (46.5%)	15 (15.5%)	**<0.001**
TIPI extraversion, n (%)	688 (58.3%)	495 (58.7%)	193 (57.1%)	
Mean ± SD (range)	4.1 ± 1.5 (1-7)	4.0 ± 1.5 (1-7)	4.2 ± 1.6 (1-7)	**0.041**
Highly extraverted, n (%)	102 (14.8%)	64 (12.9%)	38 (19.7%)	**0.025**
TIPI agreeableness, n (%)	686 (58.1%)	491 (58.2%)	195 (57.7%)	
Mean ± SD (range)	5.1 ± 1.3 (1-7)	5.0 ± 1.3 (1-7)	5.2 ± 1.2 (1.5-7)	0.20
High agreeableness, n, (%)	83 (12.1%)	66 (13.4%)	17 (8.7%)	0.09
TIPI conscientiousness, n (%)	692 (58.6%)	497 (59%)	195 (57.7%)	
Mean ± SD (range)	5.6 ± 1.3 (1-7)	5.6 ± 1.3 (1-7)	5.7 ± 1.2 (2-7)	0.28
High conscientiousness, n (%)	166 (24.0%)	118 (23.7%)	48 (24.6%)	0.81
TIPI emotional stability, n (%)	694 (58.8%)	498 (59%)	196 (58%)	
Mean ± SD (range)	5.0 ± 1.5 (1-7)	4.9 ± 1.5 (1-7)	5.2 ± 1.5 (1-7)	**0.009**
High emotional stability, n (%)	188 (27.1%)	128 (25.7%)	60 (30.6%)	0.19
TIPI openness, n (%)	693 (58.7%)	498 (59%)	195 (57.7%)	
Mean ± SD (range)	4.9 ± 1.4 (1-7)	4.9 ± 1.4 (1-7)	4.9 ± 1.2 (1-7)	0.50
High openness, n (%)	55 (7.9%)	44 (8.8%)	11 (5.6%)	0.16
IPIP emotional stability, n (%)	695 (58.8%)	499 (59.2%)	196 (58%)	
Mean ± SD (range)	34.1 ± 8.9 (10-50)	33.1 ± 8.9 (10-50)	36.7 ± 8.2 (14-50)	**<0.001**
PCS, n (%) (mean ± SD, [range])	1061 (89.8%)	716 (84.9%)	300 (88.8%)	
Total	14.9 ± 13.2 (0-52)	17.7 ± 13.5 (0-52)	8.2 ± 9.7 (0-39)	**<0.001**
PCS rumination	5.4 ± 4.9 (0-16)	6.3 ± 4.9 (0-16)	3.4 ± 4.1 (0-16)	**<0.001**
PCS magnification	3.2 ± 3.0 (0-12)	3.8 ± 3.1 (0-12)	1.8 ± 2.2 (0-11)	**<0.001**
PCS helplessness	6.4 ± 6.3 (0-24)	7.7 ± 6.5 (0-24)	3.0 ± 4.2 (0-19)	**<0.001**

* Defined as more than 7 drinks per week of 0.5 to 0.6 L beer or 0.25 to 0.3 L wine or 25 mL spirits for males and more than 3.5 drinks/week of 0.25 to 0.3 L beer or 0.125 to 0.15 L wine or 12.5 mL spirits for females.

IPIP, The 10-item International Personality Item Pool's; PCS, Pain Catastrophizing Scale; PROMIS, Patient-Reported Outcomes Measurement Information System; TIPI, Ten-Item Personality Inventory.

*P* (significance) refers to painful vs painless neuropathy.

The most affected area (99.5% of patients) was the feet. Most of the patients were Caucasian (99.1%) and suffered from diabetic neuropathy (73.8%).

According to the QST, the majority of patients was allocated to the sensory loss phenotype (>50%), followed by the mechanical hyperalgesia phenotype in app. 26% and the thermal hyperalgesia phenotype in app. 19%.

### 3.1. Painful neuropathy

Pain characteristics of patients with painful neuropathy are shown in Table 1. More than 80% had experienced pain for at least 1 year with a high number of patients being only minor impaired according to the CPG (app. 45%, CPG 1 and 2, Table 1). More than 60% were living with persistent pain with or without paroxysmal attacks (Table 1). About 56.1% of patients reported taking pain medication. Of these, 78.2% were treated according to current treatment recommendations for neuropathic pain,^22^ ie, had anticonvulsants, antidepressants, topicals, or opioids.

### 3.2. Painless neuropathy

Thirty-seven (app. 11%) of the patients classified as painless reported to suffer from pain of other origin. Of these, 81% reported that they had not suffered from pain within the last 24 hours (BPI), and 77.7% reported that they had not suffered from pain within the last 7 days (BPI) or 4 weeks (BPI). Mean pain intensities were 0.54 ± 1.2, range 0 to 5 NRS (numeric rating scale with zero meaning “no pain” and 10 meaning “the worst pain imaginable) for pain within the last 24 hours; 0.6 ± 1.6, range 0 to 6 NRS for pain intensity within the last 7 days; and 0.75 ± 1.7, range 0 to 9 NRS for pain intensity with the last 4 weeks in the group with painless neuropathy.

### 3.3. Comparison of patients with painful and painless neuropathy

Compared with painless patients, those reporting painful neuropathy were younger, had a higher BMI, a more severe neuropathy, and were less likely to have diabetic neuropathy and more likely to have idiopathic neuropathy. They also had a higher frequency of pain in their family, higher depression and anxiety scores, more sleep problems and fatigue, were less extraverted and emotional stable, and showed higher levels of pain-related worrying. Finally, they also reported less current or previous misuse of alcohol and a higher number of packyears of smoking compared to patients with painless neuropathy (Tables 2 and 3). The frequency of the different sensory clusters (loss, mechanical and thermal hyperalgesia) was similar in both groups (Table 2).

### 3.4. Risk estimation for painful neuropathy using multivariate logistic regression

Multivariate logistic regression with MICE demonstrated that neuropathy severity (TCSS total score, *P* < 0.0001), etiology of neuropathy (presence of idiopathic neuropathy including patients with only small-fiber neuropathy, *P* < 0.0001), presence of chronic pain in family (*P* < 0.0001), PROMIS Depression (*P* = 0.0383), Fatigue T-Score (*P* = 0.005), and PCS total score (*P* = 0.0003) were the most important parameters associated with the presence of pain in neuropathy (Table 4).

**Table 4:** Final results of the multivariate logistic regression with multivariate imputation by chained equations.

	Coefficient	*P*
Presence of chronic pain in family	1.2464	**<**0.0001
PROMIS depression T score	0.0304	**0.0383**
PROMIS fatigue T score	**0.0454**	**0.0051**
PCS total score	**0.0334**	**0.0003**
Toronto total score	**0.109**	**<0.0001**
Diagnosis of neuropathy: chemotherapy induced	−0.0755	0.8254
Diagnosis of neuropathy: idiopathic	**1.4973**	**<0.0001**
Diagnosis of neuropathy: other*	0.8155	0.1326

This table show the regression coefficients and their respective *P*-values of all variables included in the multivariate logistic regression model investigating their influence on pain status (painful or painless neuropathy). A positive coefficient represents an increased probability of painful neuropathy with increasing value of the respective variable.

Note that chemotherapy-induced neuropathy has a negative algebraic sign, meaning that this is not a risk factor for painful neuropathy but rather induced painless neuropathy in our sample.

* Diagnosis of neuropathy “other” includes alcoholic neuropathy, Vit. B12-deficiency, hereditary.

IPIP, International Personality Item Pool's; MICE, multivariate imputation by chained equations; PROMIS, Patient-Reported Outcomes Measurement Information System; PCS, Pain Catastrophizing Scale; TIPI, Ten-Item Personality Inventory.

The results for the 4 performance metrics, accuracy, balanced accuracy, F1, and Kappa, are illustrated in Table 5. Using the logistic regression for predicting painful or painless neuropathy, an accuracy of 78% is achieved, which means that almost 4 out of 5 patients will be classified correctly.

**Table 5:** Final results of the logistic regression and random forest analysis by comparison.

	Logistic regression	Random forest
Accuracy	0.7772	0.7612
Kappa	0.4094	0.4082
Balanced accuracy	0.6891	0.7016
F1	0.5536	0.5740

This tables demonstrates and compares the performance of the 2 statistical analyses applied to classify pain status (painless or painful neuropathy). Because of imbalance of the classes in addition to the accuracy (overall relative frequency of correct classifications) and Kappa-statistics, the balanced accuracy (arithmetic mean of relative frequencies of correct classifications of both classes) and F1 statistics are shown (see Methods section for details). Higher values indicate better performance, max. value is 1 (100%).

### 3.5. Risk estimation for painful neuropathy using machine learning

**Figure 2.**
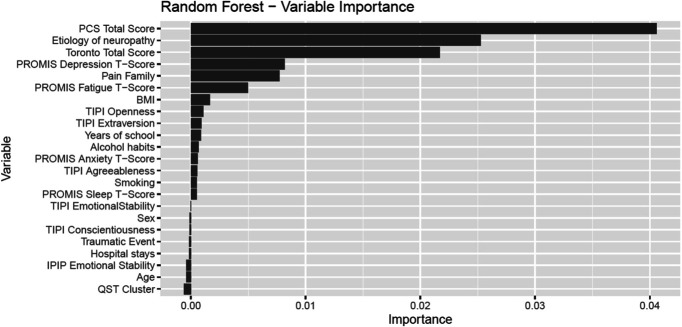
Importance of variables for the presence of painful neuropathy. This figure illustrates the variable importance (VI) of variables included in the random forest analysis to classify pain status (painless or painful neuropathy), ranked from highest importance to lowest. Higher VI demonstrates a stronger contribution to the accuracy (relative frequency of correct classifications) and thus a higher relevance for classifying pain status compared to variables with a lower VI.

Random forest revealed almost the same variables accounting for the presence of painful neuropathy compared to MLR suggesting that results of this analysis are robust (Fig. 2).

Using random forest with 500 trees for predicting painful or painless neuropathy, an accuracy of 76% is achieved; thus, more than 3 out of 4 patients will be classified correctly (Table 5). The analysis on variable importance also determined the PCS total score, etiology of neuropathy, TCSS total score, presence of chronic pain in family, PROMIS Depression, and PROMIS Fatigue T-Scores as the most relevant.

### 3.6. Goals and meaning of the different statistical prediction models

Among a vast variety of different candidates, the most meaningful variables for predicting painful or painless neuropathy were identified as mentioned above. Subsequently, the identified variables were combined to actually classify neuropathy (painful/painless). For these purposes, 2 different statistical models were applied for robustness.

Logistic regression models the probability of painful neuropathy in a linear fashion, allowing to express the relationship between predictors and outcome in a closed (ie, direct and explicit) form. For our reduced model, the following formula results for the probability (*P*) of a painful neuropathy:

$$P=\frac{\exp\;\left(Z\right)}{\exp\left(1+Z\right)},\text{with}$$

Z=−4.81+1.25PainFamily+0.03×PROMISDepression+0.045×PROMISFatigue+0.033PCS+0.109TTS−0.076×Etiology of neuropathy:Chemotherapy−induced+1.50×Etiology of neuropathy:idiopathic+0.82×Etiology of neuropathy:other

History of chronic pain in family are coded No = 0, Yes = 1. Etiology of neuropathy is dummy-coded; thus, if neither chemotherapy-induced, idiopathic or other is true, a 0 has to be inserted for all 3 variables. For example, a subject with presence of chronic pain in the family (PainFamily = 1), a PROMIS Depression and Fatigue score of 55 and 51, respectively, a PCS total score of 20, a TCSS of 11, and a chemotherapy-induced neuropathy (thus the other dummy-variables 0) has an estimated probability of a painful neuropathy of 91.1%.

Random forest generates 500 decision trees based on the described random samples of observations. Future subjects follow the branches of all 500 trees according to their individual patient characteristics. For example, a decision node of a tree might check whether “PCS total score <15.” If this is true, the subject follows the left branch; if false, the right branch and continue to the next decision node in that tree. Each subject thus ends up in 500, potentially different, final nodes, so-called “leafs.” Each leaf represents a classification for painless or painful neuropathy. The overall final classification is made by a majority vote. Variables meaningful for the prediction are identified by their importance regarding all 500 trees.

Thus, both procedures fulfil both goals of identifying meaningful variables and allowing to predict the outcome, ie, painful or painless neuropathy, based on the identified variables.

## 4. Discussion

We conducted a multicenter, cross-sectional analysis integrating and investigating clinical and sensory phenotype as well as extensive and detailed demographic and family data, personality, individual differences, emotional well-being, and life-style variables for the prediction of painful neuropathy. Both analyses, MLR and ML, revealed that neuropathy severity and idiopathic etiology as well as family history of pain, pain-related worrying, depressed mood, and fatigue are associated with painful neuropathy.

While logistic regression models the probability of painful neuropathy in a linear fashion, allowing to express the relationship between predictors and outcome in a closed (ie, direct and explicit) form, random forest generates 500 decision trees based on the described random samples of observations. Future subjects follow the branches of all 500 trees according to their individual patient characteristics. For example, a decision node of a tree might check whether “PCS total score <15.” If this is true, the subject follows the left branch; if false, the right branch and continue to the next decision node in that tree. Each subject thus ends up in 500, potentially different, final nodes, so-called “leafs.” Each leaf represents a classification for painless or painful neuropathy. The overall final classification is made by a majority vote. Variables meaningful for the prediction are identified by their importance regarding all 500 trees. Thus, both procedures fulfil both goals of identifying meaningful variables and allowing to predict the outcome, ie, painful or painless neuropathy, based on the identified variables.

In the present study, polyneuropathy was defined using the criteria according to the Toronto Consensus Panel on Diabetic Neuropathy,^63^ neuropathic pain according to the NeuPSIG definitions,^23^ and the TCSS^10^ for severity of neuropathy. As various clinician-rated measures that incorporate neuropathy signs exist, it is not clear which of these measures performs best for various polyneuropathy phenotypes.^24^ Results might have been different if other classifications would have been used.

Some of the factors associated with painful neuropathy have already been shown to be risk factors associated with chronic pain or are well known to modulate pain perception such as pain-related worrying and emotional well-being,^27,44,54,61^ larger areas of hypoesthesia after surgery,^2^ family history of body pain,^76^ or obesity.^60,61^ A unique feature of this study, however, is the integration of sensory phenotype as well as extensive and detailed demographic and family data, personality, emotional well-being, and life-style variables. Our analysis identified some unique factors associated with painful neuropathy. Although the use of a cross-sectional design does not allow cause–effect inferences, our findings may provide input to research specifically focusing upon causal inference.^17^ These results may then help to identifying patients at risk for the development of painful neuropathy in clinical practice. Our findings are promising in this regard: our final algorithms allow to classify patients with painful neuropathy in almost 4 out of 5 cases.

Supporting our findings, a recent study testing ML approaches including random forest applied to questionnaire data only in people with diabetic neuropathy also found that depression and personality traits were of value in classifying painful (vs painless) diabetic neuropathy in addition to the metabolic factors HBA1C and BMI.^7^

In this study, factors from each dimension of the biopsychosocial model of pain were associated with neuropathic pain: biological factors (severity of neuropathy, etiology), psychological factors (depression, fatigue, anxiety, pain-related worrying), as well as social factors (pain in family). This strengthens the importance to not only focus on one of these 3 factors in the risk evaluation for chronic pain, ie, to consider the patient not only in terms of the biological causes of pain, but also in terms of his or her emotional state and social environment. This might be useful not only for diagnostic settings but also for therapy evaluation.^26,49^

Comparison of patients with painful and painless neuropathy revealed that patients with painful neuropathy were slightly younger and not as extraverted and emotional stable than patients with painless neuropathy. Often older age is thought to be associated with a risk for the development of chronic pain such as in postherpetic neuralgia,^30,38,73^ but there are also several prospective and retrospective studies that have demonstrated that younger age is associated with the presence of chronic pain.^35,52,72^ The exact mechanisms how age influences pain are still unclear. While a reduced immune system in the elderly and age-related changes of the nociceptive system because of, for example, a diminished supply of neurotrophic factors and thus a higher susceptibility to neuropathy-inducing insults might add to the risk for postherpetic neuralgia in older people,^51^ other factors, ie, psychological or social might be more important than biological reasons for the risk of chronic pain in younger patients. This again strengthens the importance to regard pain in a biopsychosocial context. However, it is noteworthy that age did not turn out to be an important predictor in the final multivariate model.

Of note, almost half of the patients with painful neuropathy reported low levels of pain-related disability measured with CPG. This fits well with the observation that the influence on daily life does not result from CPG or pain intensity.^25^

Interestingly, in this study, diabetes did not turn out as a risk factor for painful neuropathy—the frequency of diabetic neuropathy was even lower than in patients with painless neuropathy. Unfortunately, we did not ask for previous pain conditions during the course of diabetes. It might be possible that painful neuropathy in some patients moved to painless neuropathy in the course of disease. However, this can only be investigated in prospective longitudinal studies and not in a cross-sectional study like the one performed here. So far, few longitudinal studies are available; one recently published study interestingly showed an increased number of patients with pain in diabetic neuropathy upon 5-year follow-up.^77^ Thus, it seems also necessary for future studies to investigate stability of clinical phenotypes over the years.

## 5. Limitations

Our aim was to investigate the factors that are associated with painful neuropathy and not for the occurrence of pain overall. Our categorization of painful and painless neuropathy (see methods) did not take into account other forms of pain. It may well be that these other forms of pain may have impacted our results. Pain, regardless of whether it is neuropathic or not,^43^ has an impact of mood and quality of life. Nevertheless, we attempted to minimize that bias by excluding patients with other pain conditions in the area of neuropathy. Also, all included patients were instructed to fill out the questionnaires with regard to the symptoms of neuropathy.

Our study design was cross-sectional, and it may well be that some factors may be consequences rather than antecedents of chronic pain, eg, depressed mood and pain-related worrying. Results should be interpreted with care and confirmed in an independent prospective longitudinal study, taking into account possible biases. This, however, is difficult in polyneuropathy because with the exception of chemotherapy-induced neuropathy, where a possible trigger of neuropathy allows for prospective investigation, the onset of neuropathy is often insidious and no starting point can be determined. Additionally, even in chemotherapy-induced neuropathy, prior existing risk factors for a manifestation of neuropathy have been demonstrated such as obesity, smoking, alcohol, nutritional deficiencies, and a poorly controlled diabetes,^34,42^ ie, here too there are individual differences present before an identical possible trigger of a neuropathy, which makes a comparison of patients difficult.

Furthermore, the participating centers in this study are specialized in pain treatment, and since about 2 thirds of the patients in this study had a painful neuropathy, there is a potential recruitment bias.

Regarding family history of chronic pain, we accept that there may be a reporting bias as we did not have the capacity to independently corroborate with the affected family member unless they had been independently recruited to DOLORisk.

Additionally, some etiologies of neuropathy, eg, diabetes, might be more severely affected in health status because of the numerous body systems affected by diabetes, which might distort the results. Furthermore, we cannot exclude an influence of medication on questionnaire results such as, for example, treatment with antidepressants.

## 6. Conclusion

The knowledge of associated factors for chronic neuropathic pain is important. Neuropathic pain is likely to arise from a complex interaction of biopsychosocial factors. We have made a first step to predict the probability of painful neuropathy classification through multiple statistical analyses. Knowing individual risk might help in the future to prevent the onset of pain by early interventions.

Further multicenter and longitudinal prospective studies are necessary to confirm our results and understand the causal relationships between the factors that we have identified in this cross-sectional cohort and neuropathic pain development and progression.

## Conflict of interest statement

A.S.C.R. reports the following interests occurring in last 24 months: A.S.C.R. undertakes consultancy and advisory board work for Imperial College Consultants—A.S.C.R. undertakes consultancy and advisory board work for Imperial College Consultants—in the last 36 months, this has included remunerated work for Abide, Confo, Vertex, Pharmanovo, Lateral, Novartis, Mundipharma, Orion, Shanghai SIMR BiotechAsahi Kasei, Toray & Theranexis. A.S.C.R. was the owner of share options in Spinifex Pharmaceuticals from which personal benefit accrued upon the acquisition of Spinifex by Novartis in July 2015. The final payment was made in 2019. A.S.C.R. is named as an inventor on patents: A.S.C.R., Vandevoorde S., and Lambert D.M Methods using N-(2-propenyl)hexadecanamide and related amides to relieve pain. WO 2005/079771. Okuse K et al. Methods of treating pain by inhibition of vgf activity EP13702262.0/WO2013 110945. National Institute for Health Research (NIHR)—Chair of the Trial Steering Committee (TSC) for the OPTION-DM trial. Advisor British National Formulary. Member Joint Committee on Vaccine and Immunisation-varicella subcommittee. Analgesic Clinical Trial Translation: Innovations, Opportunities, and Networks (ACTTION) steering committee member. Non-Freezing Cold Injury Independent Senior Advisory Committee (NISAC): Member. Medicines and Healthcare products Regulatory Agency (MHRA), Commission on Human Medicines—Neurology, Pain & Psychiatry Expert Advisory Group.

D.B. has acted as a consultant on behalf of Oxford University Innovation in the last 36 months: Aditum Bio, Amgen, Biointervene, Bristows, LatigoBio, GSK, Ionis, Lexicon therapeutics, Lilly, Neuvati, Olipass, Orion, Regeneron, Replay, Theranexus, Third Rock ventures, Vida Ventures. He is a member of the Non-Freezing Cold Injury Independent Senior Advisory Committee (NISAC). D.B. has a patent application “a method for the treatment or prevention of pain, or excessive neuronal activity, or epilepsy” Application No. 16/337,428.

D.L.B. and A.C.T are members of the DOLORisk consortium funded by the European Commission Horizon 2020 Framework Programme (ID633491); the International Diabetic Neuropathy Consortium (IDNC) research programme, which is supported by a Novo Nordisk Foundation Challenge Programme grant (Grant number NNF14OC0011633); and PAINSTORM consortium funded by UKRI and Versus Arthritis (MR/W002388/1). D.L.H.B. is a Wellcome Investigator (202747/Z/16/Z and 223149/Z/21/Z). A.C.T. is supported by Academy of Medical Sciences Starter Grant SGL022\1086 and is an Honorary Senior Research Fellow and Carnegie-Wits Diaspora Fellow at the Brain Function Research Group, School of Physiology, Faculty of Health Sciences, University of the Witwatersrand, Johannesburg, South Africa. M.M.V.P. is a member of the DOLORisk consortium (Horizon 2020 grant agreement 633491) and the PAINSTORM consortium funded by UKRI and Versus Arthritis (MR/W002388/1).

N.A. has received honoraria from Pfizer (Advance program), Biogen, Merz, Grunenthal, Novartis, Air Liquide and Upsa over the past 36 months for consultancy or speakers bureau and is member of the Dolorisk consortium.

Outside the submitted word, N.B.F. has received consultancy fees from Vertex, Novartis Pharma, NeuroPN, Nanobiotix, Neurvati, and Samiona and has undertaken consultancy work for Aarhus University with remunerated work for Biogen, Merz, and Confo Therapeutics. She has received grants from IMI2PainCare an EU IMI 2 (Innovative medicines initiative) public-private consortium and the companies involved are Grunenthal, Bayer, Eli Lilly, Esteve, and Teva. N.B.F. is member of the Dolorisk consortium and her research is supported by the Lundbeck Foundation (R359-2020-2620).

R.B. reports the following COIs: Grant/Research support: EU Projects: “Europain” (115007). DOLORisk (633491). IMI Paincare (777500). German Federal Ministry of Education and Research (BMBF): Verbundprojekt: Frühdetektion von Schmerzchronifizierung (NoChro) (13 GW0338C). German Research Network on Neuropathic Pain (01EM0903). Pfizer Pharma GmbH, Grünenthal GmbH, Mundipharma Research GmbH und Co. KG., Alnylam Pharmaceuticals, Inc, Zambon GmbH, Sanofi Aventis GmbH. Speaker: Pfizer Pharma GmbH, Sanofi Aventis GmbH, Grünenthal GmbH, Mundipharma, Lilly GmbH, Desitin Arzneimittel GmbH, Teva GmbH, Bayer AG, MSD GmbH, Seqirus Australia Pty. Ltd, Novartis Pharma GmbH, TAD Pharma GmbH, Grünenthal SA Portugal, Grünenthal Pharma AG Schweiz, Grünenthal B.V. Niederlande, Evapharma, Takeda Pharmaceuticals International AG Schweiz, Ology Medical Education Netherlands, Ever Pharma GmbH, Amicus Therapeutics GmbH, Novo Nordisk Pharma GmbH, Chiesi GmbH, Stada Mena DWC LLC Dubai, Hexal AG, Viatris, AstraZeneca GmbH, Sandoz. Consultant: Pfizer Pharma GmbH, Sanofi Aventis GmbH, Grünenthal GmbH, Lilly, Novartis Pharma GmbH, Bristol-Myers Squibb, Biogenidec, AstraZeneca GmbH, Daiichi Sankyo, Glenmark Pharmaceuticals S.A., Seqirus Australia Pty. Ltd, Teva Pharmaceuticals Europe Niederlande, Teva GmbH, Genentech, Mundipharma International Ltd., United Kingdom, Galapagos NV, Kyowa Kirin GmbH, Vertex Pharmaceuticals, Inc, Biotest AG, Celgene GmbH, Desitin Arzneimittel GmbH, Regeneron Pharmaceuticals, Inc, USA, Theranexus DSV CEA Frankreich, Abbott Products Operations AG Schweiz, Bayer AG, Grünenthal Pharma AG Schweiz, Akcea Therapeutics Germany GmbH, Asahi Kasei Pharma Corporation, AbbVie Deutschland GmbH & Co. KG, Air Liquide Sante International Frankreich, Alnylam Germany GmbH, Lateral Pharma Pty Ltd, Hexal AG, Angelini, Janssen, SIMR Biotech Pty Ltd Australien, Confo Therapeutics N. V. Belgium, Merz Pharmaceuticals GmbH, Neumentum Inc., F. Hoffmann-La Roche Ltd. Switzerland, AlgoTherapeutix SAS France, Nanobiotix SA France, AmacaThera Inc. Canada, Heat2Move, Resano GmbH, Esteve Pharmaceuticals SA.

This work has been presented on the German Pain Congress 2022 and the World Congress of the International Association for the study of Pain 2022. The remaining authors have no conflicts of interest to declare.

Individual level data cannot be shared outside the DOLORisk consortium due to data protection reasons but derived data can be shared on request to the corresponding author (J.G.).
